# Assessing the Accuracy of Leg Mounted Sensors for Recording Dairy Cow Behavioural Activity at Pasture, in Cubicle Housing and a Straw Yard

**DOI:** 10.3390/ani12050638

**Published:** 2022-03-03

**Authors:** Gemma Charlton, Carrie Gauld, Fabio Veronesi, Steven Mark Rutter, Emma Bleach

**Affiliations:** 1 Animal Health, Behaviour and Welfare Department, Harper Adams University, Newport TF10 8NB, UK; smrutter@harper-adams.ac.uk; 2 Farm Department, Harper Adams University, Newport TF10 8NB, UK; cgauld@harper-adams.ac.uk; 3 Rezatec–Quad One, Becquerel Ave, Didcot OX11 0RA, UK; f.veronesi@gmail.com; 4 Agriculture and Environment Department, Harper Adams University, Newport TF10 8NB, UK; ebleach@harper-adams.ac.uk

**Keywords:** dairy cow, accelerometer, validation, pasture, indoor housing, straw yard

## Abstract

**Simple Summary:**

Sensors attached to the legs of cows are used to record behaviours such as lying times and step count. It is important that the information recorded by sensors is accurate, as changes to behaviour could indicate to the farmer that a cow is sick or in heat. Therefore, this study compared the behaviours recorded by IceQube sensors to those visually observed by humans, when the cows where housed at pasture, in a straw yard and in cubicle housing. The results showed that lying and standing times, the number of times the cows stood up and laid down and the number of steps recorded by the sensors and human observers was similar. Sensors accurately recording cow activity could potentially be worn by cows throughout their lives and could be used to predict and detect sick cows. This could allow the farmer to respond quicker and treat sick cows sooner, increasing animal welfare.

**Abstract:**

The accuracy of CowAlert IceQube sensors (IceRobotics Ltd., Edinburgh, UK) for recording lying duration, standing and lying transitions and number of steps when dairy cows where at pasture, in cubicle housing and in a straw yard, was investigated. Holstein Friesian cows at Harper Adams University, UK were fitted with IceQube sensors; one on the back left (BL) leg and one on the front left (FL) leg. Cows at pasture (*n* = 48), in cubicle housing (*n* = 46) and in a straw yard (*n* = 45) were visually observed. Data were analysed in two stages: (1) an initial exploratory phase determined the correlation level between sensor measurements andvisual observations. Subsequently, (2) a mixed effects modelling framework was used to check whether sensors provide significantly different measures of cow’s activities compared to the observations. Results indicate that lying and standing times are similar between the observed and recorded times, in all three locations. In terms of sensor placement, significant differences were found between the number of steps recorded between BL and FL on straw and pasture, but all other activities were similar, in each location. The accuracy of CowAlert IceQube sensors on the BL leg gives them the potential to be used as lifelong sensors.

## 1. Introduction

Cattle behaviour is influenced by the productivity, reproductive and disease state of the animal [1,2,3]. Therefore, changes in the behaviour of cattle can be used to assist in predicting and detecting health problems in dairy cattle [4]. For example, lying time is altered by lameness [5,6]. Ito et al. found that severely lame cows spent longer lying down and had longer duration of lying bouts compared to cows that were not severely lame [6], while the study of Blackie et al. suggests that more subtle changes in gait may be detected by changes in lying times [5]. Prior to calving, cows that subsequently suffered dystocia reduced their transitions from standing to lying positions more frequently than cows with eutocia [7]. In addition, mastitic cows had reduced lying times, a higher number of daily lying bouts and took more steps than control cows [8]. Furthermore, cows with hock and knee injuries lay down for less time each day than cows without lesions [9] and cows with Johnes disease lie down for less time [10]. 

Behavioural data have traditionally been collected using direct visual observations or from video recordings which are subsequently analysed. However, visual behaviour observations are time consuming, labour intensive, subjective and open to interpretation [4]. Sensors can record lying and standing, movement and stepping behaviour. They allow the behaviour of the complete herd to be monitored 24 h/d, over long periods of time, non-invasively and without affecting normal behaviour [11,12]. Their use is increasing by both researchers and producers. With increasing herd sizes, it can be difficult for producers to allocate sufficient time to observe their cattle [13], but technologies can assist in identifying individual cows in need of attention and monitoring their behaviour, health and welfare. 

When using technologies for behavioural research or as an on-farm monitoring tool, it is essential to ensure the integrity of the data collected. Several leg-based sensors which record lying behaviours of dairy cattle have been validated by comparing the data output from the sensor to live observations or video recordings [11,14]. CowAlert IceQube^®^ accelerometer-based leg sensors (IceRobotics Ltd., Edinburgh, UK) are a commercially available device intended for use as a fertility and health monitoring system for dairy herds. They have been previously validated for use on the back legs of cows in a pasture-based automatic milking system, with moderate to strong correlations observed between the IceQube and visual observations for walking, standing and lying behaviour [15]. Kok et al. validated the use of IceQubes when cows were housed with cubicles, but used a 2-sensor approach on both back legs to compare the lying bout duration and frequency recorded by these accelerometers rather than using visual observations [16]. Borchers et al. compared the lying duration from IceQubes on one back leg to visual observations when cows were in a cubicle housing system and reported strong correlations [17]. 

To date, research validating IceQubes has focused on back leg use. There has been no research validating the front leg or comparing the data output from the back to the front leg. In addition, validation studies tend to focus on sensor use in just one system, for example, at pasture or in cubicle housing. Therefore, the effect of housing environment on the integrity of sensor data has not yet been studied. Yet, it is important as sensors are increasing being used by researchers, and commercially they are becoming popular as a lifelong monitoring tool [18]. Therefore, it is important they are validated in various locations as the environment of dairy cows may change depending on age, stage of lactation or season. For example, cows may be housed indoors during the winter months, and allowed access to pasture during the summer [19]. Straw-bedded loose yards are also common in the UK, particularly for use around parturition or for sick cows [20]. If these devices are validated for different housing systems, then their scope for research or commercial use is increased.

The objectives of this study were to validate the use of CowAlert IceQube sensors for recording lying duration, lying and standing transitions and step count of dairy cows housed indoors in cubicle housing, indoors in a straw-bedded loose yard and at pasture. The second objective was to determine whether there were any differences in behaviour recorded by IceQubes on the back left (BL) and front left (FL) leg, in each of the three locations.

## 2. Materials and Methods

### 2.1. Animals and Management

As part of a long-term study investigating the activity of Holstein Friesian cows throughout their production cycle, IceQube^®^ accelerometer-based sensors (IceRobotics Ltd., Edinburgh, UK) were attached to both their back left (BL) and front left (FL) leg, using a Velcro hook and loop strap. The sensors recorded the timing, frequency and duration of standing and lying bouts and step count. Animal behaviour data, stored within the IceQube in 15-min summaries were automatically downloaded wirelessly to the CowAlert system (IceRobotics Ltd., Edinburgh, UK) twice daily each time the cows walked past the reader at the entrance to the milking parlour. For each IceQube sensor, data were provided in a spreadsheet with total lying time, standing time, number of steps and number of transitions for each 15-min time block. 

The cows were managed within a 400-cow commercial, year-round calving dairy herd at Harper Adams University, UK with their housing type based on stage of lactation and season. During the winter months, (November to April) the cows where housed indoors with 1.3 m × 2.5 m cubicles with 3 cm thick rubber mattresses. Cubicles were bedded twice weekly with sawdust and the passageways were scraped five times a day using automatic scrapers. A fresh total mixed ration ((TMR); maize silage, lucerne, wheat straw, spey syrup, sweet starch, soya hulls, minerals, limestone and urea) was provided daily at approximately 06:00 h and was pushed up several times a day. During the summer months (May to October), from approximately 09:00 h to 15:00 h the cows had access to pasture (a 1.5 ha field consisting predominately of perennial rye grass (Lolilum perenne)), however they could return to the indoor housing to access the TMR, but not the cubicles. Cows were housed in a straw yard with fresh bedding provided daily for approximately three weeks following calving. The yard was approximately 52.0 m × 13.0 m with 52.0 m × 8.8 m deep-bedded straw and a concrete floored passage (52.0 m × 4.2 m) behind the feed barrier where the cows could access TMR, provided daily at approximately 06:00 h and pushed up several times a day. An automatic scraper cleaned the passage five times a day. In all locations, the cows had ad libitum access to drinking water. Twice a day from 05:00 h and 15:00 h the cows were milked in a 40-point internal rotary parlour. Ethical approval for the study was given by Harper Adams University Research Ethics Committee (project number -5647-201506).

### 2.2. Behavioural Recordings

Four observers were trained to perform live visual observations, and inter-observer reliability was assessed as follows. During training, the observers became familiar with the behavioural ethograms (Table 1 and Table 2) and following on-farm discussions and examples shown of each behaviour, the observers carried out a 2 h observation on the same focal animal. Observers did not share or discuss the behaviours they recorded. Following the session, the agreement between each observer and the lead author was assessed to ensure high repeatability. 

At pasture, 48 cows were visually observed between August and September 2015. In the straw yard, 45 cows were observed between August and December 2015 and in the cubicle housing 46 cows were observed between January and April 2016. In total, 139 cows were continuously observed for a 2 h period between 09:00 h and 15:00 h by one of the trained observers. The number of cows observed and the number of observers recording cow behaviour varied each day. The posture of the cow was recorded (lying, standing, walking; Table 1) as well as step count for the front and back left leg. A step was defined as ‘lifting the hoof off the ground and placing it in front of the original placement’. Stopwatches (ASW01; PeersHardy Ltd., Birmingham, UK) were used to record the time of each leg movement or posture change and the duration of behaviours during each observation block and had their time synchronized with the IceManager software, used to programme the IceQubesThe time recorded during visual observations was used to match the observation and IceQube activity data and the results were compared. 

### 2.3. Statistical Analysis 

IceQube data were provided in 15-min blocks, so 1 h of visual observation data were matched by time period to the four corresponding blocks of IceQube data (similar to Ungar and Rutter) [21]. Data from the IceQubes revealed several short lying and standing bouts ranging from 5–15 s. The next shortest bout was 1 min 58 s, so all bouts ≤ 15 s were removed as suggested by Mattachini et al. and Kok et al. [16,22]. 

A formal statistical model framework was used to compute statistical differences between IceQube recordings and visual observations. This model was selected as the experimental design was necessarily within-subject, since it involved recording data from both BL and FL on the same cow. Several models were fitted, one for each combination of behaviour variable and location, e.g., steps and indoor. Each of these models included as an additional random effect the cow identification in order to account for within-subject clustering. 

The concordance correlation coefficient (CCC, [23]) was calculated between IceQube recordings on both BL and FL and visual observations of step count and lying and standing times. In addition, the average difference between observed and recorded values were also calculated using the mean absolute deviation (MAD; the average of the absolute residuals between observed and recorded values) and bias (the average of the residuals). MAD gives an absolute figure that sums both negative and positive differences; this indicates how well the two measures are related. Bias gives a direction of difference, with a negative value meaning that the IceQube recorded higher values compared to visual observation. CCC were classified according to the criteria of Bikker et al. and Borchers et al.: negligible (0.00–0.30), slight (0.31–0.50), minor (0.51–0.70), moderate (0.71–0.90) and strong (0.91–1.00) [17,24]. The statistical analysis was carried out with R programming language, using the lmerTest package (version 3.1) to fit the mixed effects models and the emmeans package to compute the marginal means. *p* < 0.05 was used as the significance threshold and a trend was considered when *p* < 0.10.

## 3. Results

### 3.1. Interobserver Variability

Cohen’s Kappa revealed near perfect inter-observer agreement between the four observers (k > 0.97).

### 3.2. Indoor Cubicle Housing 

**Figure 1:**
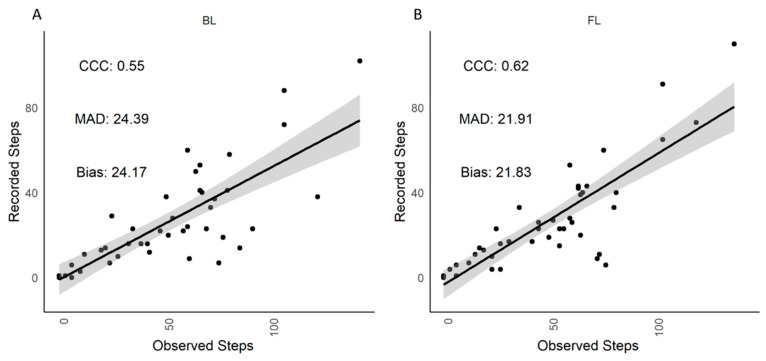
Indoor cubicle housing. Scatterplot of visually observed and IceQube recorded steps of (**A**) back left (BL) leg and (**B**) front left (FL) leg. The regression line (solid line) and confidence interval (shaded area) are presented. CCC = concordance correlation coefficient, MAD = mean absolute deviation.

Lying time (min/h), standing time (min/h) and transitions (no./h) in cubicle housing were similar (*p* > 0.05) between visual observations and recordings by the IceQubes (Table 2). There was no difference between the data recorded by IceQubes on the BL and FL legs (*p* > 0.05; Table 3) and correlations were strong (CCC > 0.91; Table 4). More (*p* = 0.0002) steps were recorded through visual observations than the IceQubes (Table 2). Table 4 shows that, compared to the observer, IceQubes on the BL recorded, on average, 24 fewer steps/h than the observer and IceQubes on the FL leg recorded, on average, 22 fewer steps/h. There was no difference in the number of steps recorded by the BL vs. FL (*p* > 0.05; Table 3) when the cows where in the cubicle housing. Correlations between the number of steps recorded in the cubicle housing by the observer and IceQubes on the BL leg (CCC = 0.55) and FL leg (CCC = 0.62) were minor (Figure 1).

### 3.3. Indoor Straw Yard

**Figure 2:**
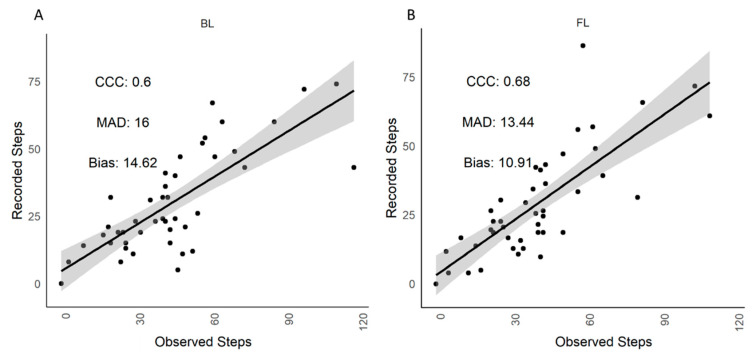
Straw yard. Scatterplot of visually observed and IceQube recorded steps in both (**A**) back left (BL) and (**B**) front left (FL) legs. The regression line (solid line) and confidence interval (shaded area) are presented. CCC = concordance correlation coefficient, MAD = mean absolute deviation.

Table 2 shows there were no differences (*p* > 0.05) in lying time (min/h), standing time (min/h) and transitions (no./h) between visual observations and the IceQube recordings when the cows were housed in the straw yard. There were also no differences (*p* > 0.05) between lying time (min/h), standing time (min/h) and transitions (no./h) recorded by IceQubes on the BL leg compared to the FL leg (Table 3) and correlations between visual observations and IceQube recordings from the BL and FL legs were strong (CCC > 0.91; Table 4). There was a difference in the number of steps recorded by visual observation and the IceQubes when the cows were in the straw yard (*p* < 0.0001; Table 2), with IceQubes on the BL leg recording, on average, 15 fewer steps than visual observations and IceQubes on the FL leg recording, on average, 11 fewer steps than visual observations (Table 4). Correlations were minor between visual observations and IceQubes on the BL leg and the FL leg (CCC = 0.60 and 0.68, respectively; Figure 2).

### 3.4. Pasture

**Figure 3:**
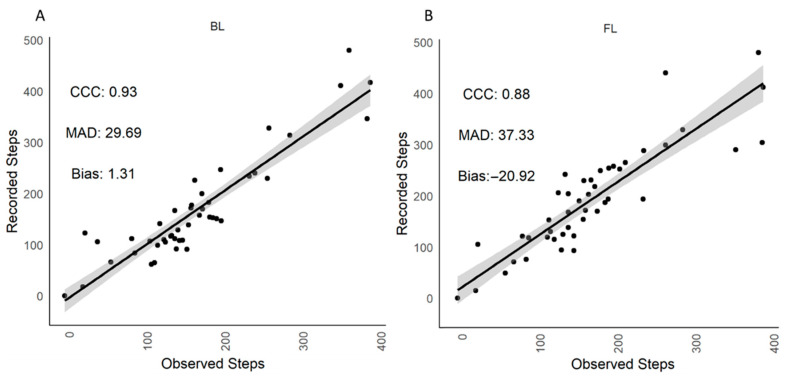
Pasture. Scatterplot of visually observed and IceQube recorded steps in both (**A**) back left (BL) and (**B**) front left (FL) legs. The regression line (solid line) and confidence interval (shaded area) are presented. CCC = concordance correlation coefficient, MAD = mean absolute deviation.

When the cows where at pasture, lying time (min/h), standing time (min/h) and number of steps/h recorded by visual observations and the IceQubes were similar (*p* > 0.05; Table 2). However, there was a difference between the number of transitions recorded (*p* = 0.009; Table 2), with the IceQubes recording more transitions than the observer (31 vs. 28 transitions/h for IceQube vs. visual observations, respectively). Table 3 shows there were no differences (*p* > 0.05) between lying time (min/h), standing time (min/h) and transitions (no./h) recorded by IceQubes on the BL vs. FL leg, however there was a difference in the number of steps recorded (*p* = 0.0005; 7509 vs. 8718 steps/h for BL vs. FL IceQube, respectively). There were strong correlations (CCC > 0.91) between visual observations and IceQubes on the BL leg for lying time (min/h), standing time (min/h), transitions (no./h) and steps (no./h; Figure 3). However, when the IceQubes were on the FL leg the correlations with visual observations, although strong for lying time (min/h) and standing time (min/h), were only moderate for transitions (no./h; CCC = 0.81) and steps (no./h; CCC = 0.88; Figure 3), with IceQubes on the FL leg recording, on average, 21 steps/h more than the visual observers (Table 4).

## 4. Discussion

Standing and lying times and transitions in all three locations, plus step count at pasture, recorded by the CowAlert IceQubes, correlated well with visual observations. The strongest correlations were found with outputs from sensors on the BL leg of cows at pasture. Although the correlations between the observer and IceQubes were minor for step count when cows were in indoor cubicle housing and in the straw yard, IceQubes on the BL leg of cows in these locations strongly correlated for lying duration, standing duration and number of transitions. 

CowAlert IceQubes have been previously validated, but the validation has generally been undertaken using cows in cubicle housing, been limited to one aspect of activity, and using IceQubes on the back leg. For example, Elischer et al. evaluated the IceQube’s ability to measure lying time compared to visual observations using a Pearson correlation coefficient and found a similar correlation to the current study (r = 0.97) [15]. Borchers et al. also compared the lying time of dairy cows in cubicle housing from the IceQube to visual observations and reported a strong correlation (r > 0.99 and CCC > 0.99) [17]. Kok et al. validated lying bouts recorded by IceQubes on dairy cows housed indoors with cubicles [16]. However, rather than comparing IceQube data to visual behaviour observations, these authors used a 2-sensor approach with the cows wearing an IceQube on each back leg and the data output from each IceQube was compared to the other, for classification of true lying bouts.

In the current study the IceQube sensors were accurate at recording lying and standing duration and the number of transitions when cows where housed in the straw yard, and they achieved a minor correlation to visual observation for step count. Although IceQubes have been compared to AfiTagII devices whilst cows where housed in deep bedded pens [25], to our knowledge, IceQubes have not previously been validated for use on dairy cows in a straw yard by comparing them to visual observations. With many dairy cows kept in loose housed systems during the peri-parturient transition period, as it provides a softer lying surface, better traction and facilitates lying and standing [26] and with investigations to determine whether leg mounted sensors can be used to predict calving increasing [27], determining their accuracy in a straw yard is important. 

At pasture, lying duration, standing duration, transitions and step count recorded by IceQubes on the BL leg were strongly correlated to visual observations. Of the 48 BL IceQubes, 8.3% (four IceQubes) overestimated the number of transitions at pasture. O’Driscoll et al. also reported a number of false lying bouts (4.8 ± 2.38 (mean ± S.D. (standard deviation))) lying bouts < 10 min/d) when data loggers were used at pasture [12]. Similarly, Rutter et al. found that using IceTags (a version of sensor from IceRobotics intended for research) at pasture overestimated the number of lying bouts by 27% [28]. These finding by Rutter et al. were based on the raw data [28], but more recent studies have suggested a threshold for lying bouts of between 25 s [22] to 33 s [16] in duration. By removing shorter lying bouts, both the accuracy and specificity improve [16]. In the current study, 14% of all IceQube devices (39 of 274) recorded an inaccurate number of transitions, and 4% of these were a result of short lying or standing bouts, with the majority < 15 s in duration. During visual observation at pasture, three lying bouts (2.8%) lasting < 15 min were observed, but such short bouts are rare. 

Step count at pasture, recorded by the BL IceQube, was strongly correlated to visual observation, and these finding are similar to Elischer et al. who reported strong correlations between observed walking and IceQube steps at pasture (Pearson correlation (Rp) = 0.90) [15]. Steps recorded at pasture were more strongly correlated than in cubicle housing and in the straw yard. This may be due to having a larger sample size of steps at pasture, or it may be a result of movement during feeding. At pasture, the cows slowly move forward as they graze and forage for food. This forward movement may be easier for the sensor to record, whereas indoors, although the cows do step and move their feet during feeding, they are not continuously moving forward as they are restricted by the feed fence, and it may be more difficult for the sensors to record this movement. 

When housed indoors, in cubicle housing, the observers recorded, on average, 23 steps/h more than the IceQubes (24 vs. 22 steps/h for BL vs. FL leg, respectively), and in the straw yard, an average of 13 more steps/h compared to the IceQube (15 vs. 11 steps/h for BL vs. FL leg, respectively). This finding may be explained as concrete flooring, typically found in indoor housing has a low coefficient of friction [29] which may have caused a change in gait, such as a decrease in stride length, to avoid the risk of slipping [30]. In the straw yard, although straw may provide better traction [26], gait may have changed, if the cows struggled to move through the straw, and this altered gait may have been difficult for the IceQubes to record. In contrast, pasture provides greater friction underfoot and allows for the expression of a normal gait [31] and this may have allowed the IceQubes on the BL leg to record the number of steps more accurately when the cows were on pasture.

IceQube sensors were designed and calibrated for use on the back leg, however this experiment verifies that apart from slightly overestimating standing and lying transitions and steps at pasture, IceQubes on the FL leg performed very similarly to those attached to the back leg, when the cows where housed in cubicle housing and in the straw yard. Borchers et al. found that Track A Cow sensors used on the front leg were strongly correlated to visual observation for recording lying time of dairy cattle in cubicle housing, however lying bout frequency and step count were not recorded [17]. Previous studies have used IceQubes on the front leg of cattle at pasture to measure daily standing and lying time and lying bouts [32,33], however, their use on the front leg was not validated by comparison with visual observations. In the current study, the slight inaccuracy of the FL leg for recording transitions at pasture may have been a result of leg position whilst lying. During observations at pasture, some cows were noted to be lying with the bottom half of their front leg almost vertical, and bent at the knee which may have caused the IceQube to record a standing position. It may also have been easier for cows to change position whilst at pasture. In addition, cows were observed running at pasture, which may have resulted in the FL IceQubes recording more steps. Neither running or the lying position observed at pasture where seen when the cows where in the cubicle housing or straw yard. 

The results from this study demonstrate that commercial animal-mounted sensors can accurately record cow behaviour and activity. Such devices could be worn by cows throughout their lives and have the potential to be used to predict and detect sick cows. This could allow the farmer to respond more quickly and treat sick cows sooner, increasing animal welfare [34].

## 5. Conclusions

These findings show that IceQube accelerometers on the back leg of dairy cows performed well in indoor cubicle housing, an indoor straw yard and at pasture, and therefore have the potential to be used as lifelong sensors in a range of dairy production systems. The IceQube was designed and calibrated for back leg use, and this experiment verifies that front leg use would marginally reduce performance when cows are at pasture. 

## Figures and Tables

**Table 1:** Ethogram for live, visual observations: cow posture [15].

Position	Description
Walk	Three or more consecutive steps made by the cow.
Stand	Body is upright, supported by at least 3 legs. Standing time officially begins when the leg bearing the pedometer is fully perpendicular (vertical) to the ground.
Lie	Body is not supported by the legs; body is in contact with the ground. Cow is resting sternally or laterally. Lying time officially begins when the leg bearing the pedometer is parallel (horizontal) to the ground.

**Table 2:** Predicted mean values (±SEM ^1^) of lying duration (min/h), standing duration (min/h), the number of lying and standing transitions (no./h) and step count (no./h) recorded by visual observation and IceQubes on the back left (BL) legs of dairy cows (*n* = 48) in indoor cubicle housing, a straw yard and at pasture.

Behaviour Variable	Visual Observations	IceQubes	*p*-Value
CUBICLE HOUSING			
Lying (min/h)	28.87 ± 2.45	29.05 ± 2.44	0.36
Standing (min/h)	18.16 ± 3.01	18.10 ± 2.89	0.93
Transition (no./h)	1.06 ± 0.09	1.12 ± 0.10	0.09
Steps (no./h)	26.08 ± 6.36	12.46 ± 3.14	0.0002
STRAW YARD			
Lying (min/h)	14.01 ± 2.89	14.33 ± 2.80	0.67
Standing (min/h)	33.91 ± 2.22	33.91 ± 2.16	0.98
Transition (no./h)	1.02 ± 0.07	1.06 ± 0.08	0.09
Steps (no./h)	36.46 ± 3.31	25.04 ± 2.34	<0.0001
PASTURE			
Lying (min/h)	21.86 ± 2.22	21.84 ± 2.22	0.87
Standing (min/h)	32.89 ± 2.37	32.91 ± 2.37	0.92
Transition (no./h)	0.86 ± 0.09	0.97 ± 0.09	0.009
Steps (no./h)	116.25 ± 20.46	123.63 ± 21.65	0.17

^1^ SEM = Standard error of the mean.

**Table 3:** Predicted mean values (±SEM ^1^) of lying duration (min/h), standing duration (min/h), the number of lying and standing transitions (no./h) and step count (no./h) recorded by IceQubes on the back left (BL) and front left (FL) legs of dairy cows (*n* = 48) in indoor cubicle housing, a straw yard and at pasture.

Behaviour Variable	BL	FL	*p*-Value
CUBICLE HOUSING			
Lying (min/h)	28.97 ± 2.44	28.96 ± 2.44	0.77
Standing (min/h)	18.12 ± 2.94	18.13 ± 2.96	0.99
Transition (no./h)	1.09 ± 0.10	1.10 ± 0.10	0.32
Steps (no./h)	19.85 ± 4.78	18.69 ± 4.61	0.21
STRAW YARD			
Lying (min/h)	14.15 ± 2.85	14.20 ± 2.88	0.95
Standing (min/h)	33.96 ± 2.18	33.85 ± 2.20	0.29
Transition (no./h)	1.04 ± 0.07	1.03 ± 0.07	0.56
Steps (no./h)	31.76 ± 2.80	29.74 ± 6.66	0.05
PASTURE			
Lying (min/h)	21.83 ± 2.21	21.87 ± 2.23	0.79
Standing (min/h)	32.89 ± 2.36	32.91 ± 2.37	0.95
Transition (no./h)	0.89 ± 0.09	0.94 ± 0.09	0.18
Steps (no./h)	113.38 ± 20.07	126.50 ± 21.83	0.0005

^1^ SEM = Standard error of the mean.

**Table 4:** Explanatory analysis results comparing visually observed and IceQube recorded values for lying duration (min/h), standing duration (min/h), the number of lying and standing transitions (no./h) and step count (no./h) of both back left leg (BL) and front left leg (FL) legs of dairy cows (*n* = 48) in indoor cubicle housing, a straw yard and at pasture. CCC (concordance correlation coefficient, Lin 1989), MAD (median absolute deviation; the average of the absolute residuals between observed and recorded values) and bias (the average of the residuals) are presented.

Behaviour Variable	CCC		MAD		Bias	
	BL	FL	BL	FL	BL	FL
CUBICLE HOUSING						
Lying (min/h)	1.00	1.00	0.91	0.96	−0.22	−0.17
Standing (min/h)	0.99	0.99	1.09	1.15	0.52	0.50
Transition (no./h)	0.95	0.93	0.04	0.07	−0.04	−0.07
Steps (no./h)	0.55	0.62	24.39	21.91	24.17	21.83
STRAW YARD						
Lying (min/h)	0.99	0.99	0.78	1.11	0.16	−0.04
Standing (min/h)	1.00	0.99	0.87	1.09	−0.11	0.02
Transition (no./h)	0.92	0.96	0.04	0.02	−0.04	−0.02
Steps (no./h)	0.60	0.68	16.00	13.44	14.62	10.91
PASTURE						
Lying (min/h)	1.00	0.99	0.46	1.27	0.08	−0.02
Standing (min/h)	1.00	0.99	0.48	1.23	−0.02	−0.02
Transition (no./h)	0.94	0.81	0.06	0.15	−0.06	−0.15
Steps (no./h)	0.93	0.88	29.69	37.33	1.31	−20.92

## Data Availability

The datasets used and analysed during the current study are available from the corresponding author on reasonable request.
